# Pathological and Practical Researches on Diseases of the Intestinal Canal

**Published:** 1829-07-01

**Authors:** 


### Medico-Chirurgical Review;

### No. XXI.

APRIL 1, to JULY 1, 1829.

### I.

**Pathological and Practical Researches on Diseases of the Stomach, the Intestinal Canal, the Liver, and other Viscera of the Abdomen. By *John Abercrombie*, M.D. Fellow of the Royal College of Physicians of Edinburgh, &c. and first Physician to His Majesty in Scotland. Octavo, pp. 396. 1828.**

In resuming our review of Dr. Abercrombie's Pathological Labours, affections of the Intestinal Canal are to occupy our attention; and, although the variety of topics embraced by our first article presented more novelty, the subject of the present is not less important. The diversity of functions allotted to the alimentary tube, and the necessity of their due and daily performance; their effects, when disordered, upon the general health, and their influence when healthy upon the progress of disease, have given to this organ a character and a rank which entitle it to much attention. Before the observations of Dr. Hamilton upon Purgatives were made public, the distant sympathies and numerous connexions of the primæ viæ were understood but little, and valued less. Regarded chiefly as an *excretory duct*, their states and sympathies were carelessly observed, and, unless they became troublesome through relaxation, or uneasy from confinement, spasm or inflammation was required to gain for them a proper share of the physician's notice. But since the appearance of that work, medicine has sustained an important change, and diseases, which were ascribed to the nervous system as their seat, and pointed to nervous remedies as their cure, are now studied in connexion with intestinal derangement, and subjected to a course of purgative medicines.

That this therapeutic innovation has done good cannot be denied; that it is grounded upon principles more reasonable in themselves and better known to us is not to be disputed; and that the attention which we paid, or rather the negligence with which we attended to the important functions of an important organ was highly culpable, none are more ready to admit; but we have for some time suspected that we are now sinning on the other side, and that the anxiety, with which we now examine every stool, and look forward to every evacuation, resembles much more devotion to an idol than sensible and enlightened knowledge. In France, this *à posteriori* system is still more popular than with us, and no one can witness, without some feeling either of ridicule or contempt, the devotion which is paid to Cloacina in the schools of Broussais. In them it seems to be a point not yet decided, whether the physician was made for the night-table or the night-table for the physician; but sure it is, that, between mucous inflammation as the disease, and leeches and purgatives as its cure, his skill is mainly, if not wholly occupied. In this remark we restrict ourselves to the adherents of Broussais—the followers of Laennec, *perhaps*, ride another hobby—and, whether the complaint be referred to the head or the toe, whether the ostensible symptoms be cerebral or thoracic, mucous action must explain all, and all must be treated by *fundamental* remedies.

It were unnecessary to observe that, like the stomach, the intestines are composed of three coats, did not their different textures and functions render them obnoxious to different forms of disease; but the symptoms of each are very much alike, and the treatment of all generally similar. From the peculiar situation of the mucous coat it is most frequently exposed to the causes of disease, and when diseased, is characterized by phenomena peculiar to itself, the most palpable of which are merely functional. It may, however, as we shall see hereafter, be very extensively disorganized without betraying *any* symptoms; and if we have any data pathognomonic of peritoneal and muscular inflammations of the fæcal tube, they are exceedingly equivocal, and require an extensive experience to render them available. Like inflammation of all other contiguous textures, the action of the one is easily transferable to the other, many of the signs of each are common to both, and a diagnosis between them must be more appreciated for nosological than practical purposes. It is, perhaps, true, as our author insists, that muscular fibre, when inflamed, often ends in gangrene, that serous texture frequently forms false membrane, and mucous tissue is disposed to end in ulceration; but it is also true that all of these terminations may be variously modified, and that each of these tissues may pass into any of these states. Thus, Dr. Pemberton observes that inflammation of the mucous coat *generally* ends by throwing out coagulable lymph, which may be discovered in the stools, resembling shreds of boiled macaroni; and it is daily seen in enteritis, dysentery, consumption and fever, that the muscular and peritoneal coats are by no means exempt, either from gangrene, or ulcerative disease. To decide, therefore, with precision upon the texture deranged, even by the nature of the post-mortem appearances, might frequently be hazardous, and if so in cases which have run their course, and in which our diagnosis is enlightened by seeing the effects of disease after death, is it not more strikingly certain in cases which are still in progress, and which furnish nothing in aid of our investigations but equivocal symptoms very imperfectly pronounced? We fear that the Dr. has on this point refined too much, when he observes that muscular inflammation is marked by obstructed bowels, and that an unaffected state of the intestinal functions characterises an inflammatory state of the peritoneal coat. Admitting with the Dr. that inflammation of the mucous coat is always marked by diarrhœa, in opposition to the authority of our best writers, our own experience, and the testimony of Broussais himself, we apprehend it will be generally allowed that peritonitis is nearly as often accompanied with constipation as enteritis, that the alvine functions may remain undisturbed in either, and that, if there be no diagnostic character between them better marked than obstruction during the disease and gangrene after death, it will prove a very fallible criterion.

"But, besides the various forms of inflammatory affections of the intestinal canal, there is a class of diseases entirely distinct, namely, that which affects it simply as a muscular organ. This includes the various modifications of ileus, which, though it very often terminates by inflammation and its consequences, is in its early stages to be considered as a disease of the canal, affecting chiefly its muscular action. The investigation of the pathology of the intestinal canal might, therefore, divide itself into diseases affecting it as a muscular organ, including the varieties of ileus,—and the inflammatory diseases under three classes; namely, 1st, Simple Peritonitis, without any derangement of the muscular action of the bowels,—2d, Peritonitis combined with obstruction of the bowels, constituting the disease commonly called Enteritis,—3d, Inflammation of the mucous membrane.

"This is perhaps the correct pathological division of the subject, but I think it will answer the purposes of practical utility to consider peritonitis and enteritis together, and the diseases of the mucous membrane separately. On this plan, the actual division of the subject will be

"1. Ileus.

"II. The inflammatory affections of the more external parts, including peritonitis and enteritis.

"III. The diseases of the mucous membrane.

"The principal organic affections, and the various forms of chronic disease of the intestinal canal, are so connected with one or other of these classes, that the consideration of them must be very much combined." 103.

### Ileus.

*Ileus,* or to use a more familiar and not less correct name, *Colic* is marked by obstinate constipation, pain of bowels, especially severe around the umbilicus, which is rather relieved than increased by pressure, and vomiting. This last symptom is not necessarily nor always present, but the two former are; and if mal-treatment or neglect permit the disease to pass beyond the first stage, the abdomen generally gets tense and tender, the vomiting stercoraceous, and the patient either sinks exhausted from severe suffering, or is cut off by inflammation.

Our author divides this affection into three forms;—the *first* he entitles *simple* ileus, or ileus occurring without any previous disease; the *second,* ileus arising from previous disease, but of such a nature as merely to derange the muscular power of the intestines; and the *third,* ileus with mechanical obstruction.

In *simple ileus* death may occur during various conditions of the affected structure. Thus, it may prove fatal while the intestine affected is only in a state of over-distention, or this dilated state may be complicated with change of colour, or gangrene may have come on, and the gangrene may be combined with inflammatory exudation. But in whatever form of the disease life be extinguished, and however the closing scene may be modified by the multiplicity or inveteracy of its symptoms, distention seems to be the first *effect* of the disease, upon which the subsequent alterations in structure and aspect mainly, if not entirely depend. When spasm has once destroyed the tone of an organ, a very moderate excitement can induce more extensive disease; and when the coats of the intestines become excessively distended, their tonicity is either injured or destroyed, and a short continuance of the cause, which at first occasioned their enlargement, will be sufficient to run them into more matured disease. To illustrate the nature and progress of ileus from simple distention to complete gangrene of the gut, we shall give a rapid sketch of four cases, the first and second of which proved fatal from exhaustion, the third and fourth from inflammation and its consequences.

A woman, aged 20, having had her menses arrested by exposure to wet, was seized with violent pain in the upper part of her abdomen, attended with obstinate costiveness and frequent vomiting. On the 24th of June the pain was excessive, hiccup frequent, no stool, pulse 88, small, severe vomiting, belly tense and tender ; on the 25th, no stool, every thing vomited, pain gone, pulse feeble; on the 26th, no stool, pain not returned, vomiting and hiccup continue ; during the night she died.

*Inspection.* Lower part of ileum and colon contracted and of a white colour, but healthy to appearance; the rest of the small intestines were uniformly and greatly distended, so much so as to be transparent, and upon their surface appeared a few patches of vivid redness ; a small abscess in the left ovarium ; other parts healthy.

A lady, aged 70, whose bowels had been confined for several days, was seized on the 4th of January with violent abdominal pain and vomiting; on the 6th, the pain was less, but vomiting continued severe, and bowels costive, pulse from 80 to 90, abdomen not tender under pressure ; on the morning of the 7th, the symptoms were unchanged, but towards evening she began to sink, and died during night.

*Inspection.* Colon full of feces, but healthy ; eighteen inches of lower end of ileum contracted and empty, the remaining part of small intestines very much distended and of a dull leaden colour, with occasional patches of a livid brown. That portion of gut next to the contracted part was darkest, being nearly black. Some disease of the serous surface of the aorta; other viscera healthy.

A boy, aged 12, was affected, on the 26th of October, with violent abdominal pain, particularly severe around the umbilicus, and urgent vomiting; bowels costive, abdomen tender, pulse 50. On the 27th, pulse 120, pain more severe, tension and tenderness greater. Bleeding reduced the pulse to 112, but no stool could be procured, the pain continued unabated, sinking commenced, and between seven and eight at night he died.

*Inspection.* Stomach healthy, small intestines distended and inflamed, right side of colon gangrenous, and its caput, having burst and discharged its contents into the peritoneal sac, was collapsed. The diseased portion had been evidently much distended before sphacelus came on, and, from having lost its contractility on that account, could not, after its rupture, resume the ordinary size. The rest of the colon was quite healthy.

A man, aged 19, was attacked, on the 17th, with violent pain around umbilicus, and incessant vomiting; abdomen hard, tense, and tumid; bowels costive, countenance anxious, pulse 84. He had been ill six days without experiencing any relief from medicine, and he was now treated with bleeding, blistering, purging, and other remedies, without effect. On the 18th, pulse 120, vomiting severe, but matter vomited not feculent. On the 19th he was better; but, on the 20th, he became worse; and, on the 21st, he died.

*Inspection.* Stomach healthy, duodenum very greatly distended, in some parts transparent, in others gangrenous, bursting when handled, and in others black. This variety of condition extended to the middle of the small intestines, where a portion, 12 inches long, was empty, contracted, and healthy, below which the same diseased appearance returned, and extended till within three inches of the cæcum, where the ileum for that space again became contracted. The gangrenous portions were glued together by extensive exudation, and the contracted portions were pervious, easily dilated, and seemed unchanged in structure. Colon healthy.

In the *second form of ileus* there is a complication of diseases, and its origin is, perhaps, more easily understood than that of the foregoing form. It is, in fact, the supervention of a new upon an old disease, or the mere consequence of structural derangement. From some cause an adhesion forms within the abdomen between parts naturally free, and this adhesion counteracts or controls the functional actions of the adhering parts, or, it may be, only predisposes these parts to the agency of stimuli, which would have exerted no effect upon them under other circumstances. Ileus is thus induced; and, from the permanent nature of the cause, its symptoms are usually severe, and its issue too frequently fatal. The Dr. adduces several examples of this variety, but, since they are all essentially alike, differing only through the difference of the adherent parts, we consider it unnecessary to occupy our space by extracting more than one as an illustration; at the same time enunciating the catalogue of the rest. In the first case there is a small adhesion of the ileum to the parietes of the pelvis; in the second, the surfaces of the caput cæcum and ascending colon were attached for about two inches; in the third, the adherent intestine had formed part of a hernial tumour, to which the patient had been long addicted; in the fourth, two turns of intestine adhered by a narrow band of membrane, about an inch in length; in the sixth, ileus was occasioned by a ligamentous band, confining to the mouth of a hernial sac a portion of the sigmoid flexure of the colon; and the 5th case we shall now detail at large.

A man, aged 60, after being afflicted for a week with the ordinary symptoms of ileus, which had resisted every plan of treatment, became much exhausted by frequent attacks of vomiting. His belly was tympanitic, his pulse varied from 108 to 116, and, although the distention of his abdomen had so increased as to equal that of a woman in the last stage of pregnancy, pressure could be borne without pain, even to the last.

On *inspection* the stomach was found small and healthy, its region being principally occupied by the sigmoid flexure of the colon, which had been so distended as to *fill half the abdomen.* The upper end of the small intestines was healthy, but the lower portion was dark, and in some points gangrenous; the rectum was collapsed and healthy; some portions of the colon were not less than six inches in diameter, but for a more minute account of its sigmoid flexure, in which were the seat and source of the disease, we shall cite the original.

"The sigmoid flexure was found to have taken a singular turn upon itself, so that what is naturally its right side lay to the left, in contact with the descending colon; and the left or ascending portion of it passed in front of this portion, and lay on the right In consequence of this transposition, the rectum, as it descended from the former, namely, what was now on the left side, passed behind the lower curve of the sigmoid flexure, where it takes the first turn from the descending colon; and the rectum itself at this part received a twist, as if half round. Exactly at the point where this twist had taken place, the distention and dark colour of the diseased intestine terminated abruptly, and the remainder of the gut became white and collapsed. At this point, however, there was no mechanical obstruction, for the part was quite pervious, and, excepting the slight twist, perfectly healthy.

"In this singular case, also, I had an opportunity of ascertaining the state of the part during life. For on the 25th, three days before the man's death, having exhausted all the usual means, I was induced to examine the rectum with a large ivory-headed probang; when I found, at a certain depth, which was afterwards seen to correspond with the point where the rectum was twisted, a very slight obstruction to the passage of the instrument, which however passed with very little difficulty, and was withdrawn without any. A piece of the intestine of an animal, tied at the end, was now carried up beyond this point, and filled, by forcibly injecting water into it. This was retained for some time in the distended state, and then slowly withdrawn; but no discharge followed, though, as I have already stated, the distended intestine contained only air and fluid feces." 116.

The *third* and last form of ileus arises from obstruction to the passage of the feces, not from spasm, or, as the Dr. would have it, paralysis of the intestines, as in the first variety; nor from mere derangement of their muscular power, as in the second; but either from such change of structure within the intestine itself, as contracts its calibre, or from such neighbouring disease as, by compression or otherwise, counteracts its natural functions. This form of ileus is infinitely the most hopeless, being almost entirely beyond the resources of art, and the highest object at which, in its treatment, we can aim, is to blunt the sting of misery which we have not power to remove.

In the first case given by our author explanatory of this form, the lady had, 27 years before her death, suffered from strangulated hernia, which ended in an artificial anus in the right groin, that continued open for a considerable time, and then gradually closed. From this period she became addicted to attacks of abdominal pain and constipation, under one of which she sunk. On examination, the lower part of ileum was found in the hernial sac, and its coats both below and above this part were thickened, and had slightly contracted the area of the gut. The second, third, and fourth cases, depended on internal hernia, the strangulation having been variously effected; the five following arose from intussusception; a large gall-stone impacted in the upper part of the ileum caused the 10th; contraction of the calibre of the intestine was the origin of the 11th, 12th, 13th, and 14th; and the last arose from strictured colon. To enter into the particulars of each case is unnecessary, yet some are so peculiarly interesting, that to pass them over with so meagre a notice would be doing injustice both to our author and our readers.

A man, aged 28, was seized with the usual symptoms of ileus on the 16th, and died on the 18th. A mass of glandular disease was found in the mesentery, to which several turns of intestine had contracted adhesions; the bore of the canal at each point of adhesion being slightly diminished. At one place a piece of intestine adhered by two different points, leaving between them a free space, into which a neighbouring portion of gut had entered, and become strangulated. The canal immediately above this strictured part was gangrenous and distended.

A boy, somewhat more than two years old, was attacked on the 7th with vomiting, pain in the lower belly and tenesmus, during which he passed some blood and bloody mucus. Pulse frequent, much restlessness, countenance anxious and depressed, but abdomen easy under pressure. On the 8th, while at stool, a bloody-looking tumour, the size of an egg, protruded through the anus, which proved to be inverted intestine, and was easily reduced, but a probang was passed up by its side to a great height without reaching the commencement of the inversion; on the following morning the child died. On *inspection*, the intussuscepted parts were found to be portions of the ileum and colon, a corresponding part of the mesentery, and some of the omentum; and the tumour, which appeared through the rectum, was the caput coli inverted. *Thirty-eight inches* were occupied by the inversion, and the included portion of the colon was of a livid colour, soft, and in some places thickened.

A man, aged 24, suffered an attack of cholera a year before his death, and became from that time liable to constipation and uneasiness in his bowels. When seen by the Dr. he was much wasted, had a small pulse, distended belly, occasional vomiting, and such constipation as often baffled the most energetic medicine. The arch of the colon was found upon *inspection* very much constricted about its centre. On the left side of the stricture a very small finger could be admitted into the opening; but on the right side there ran down across the middle of the aperture a flap of fibres, connecting the upper and lower surfaces of the mucous membrane, and leaving two small lateral openings. The colon to the left of this stricture was contracted into a cord not thicker than a finger, while that to the right was distended to upwards of twelve inches in circumference, and was impacted with feculent matter of good consistence.

The last case shall be given in the author's language:—

"A girl, aged 14, previously enjoying excellent health, was seized with symptoms of ileus on the 2d of April, 1828. She was treated in the most judicious manner by Dr. Ross, but without relief, and I saw her along with him on the 3d. The pulse was then rapid and feeble; countenance anxious and exhausted; abdomen distended, tympanitic, and tender; no stool except small discharges of white mucus; frequent vomiting. She died on the 5th.

"*Inspection.*—The whole tract of the small intestine was in the highest state of distention, and of a livid colour, with some exudation of false membrane. This state terminated abruptly at about ten inches from the lower extremity of the ileum, and the remainder of the ileum was of a healthy colour, but appeared unusually thick, firm, fleshy, and of a tortuous figure. The canal of the intestine, through this portion, was found to be narrow, tortuous, or folded, so as to be traced with difficulty. On farther examination it was discovered, that this singular mass was formed by numerous small turns of the intestine adhering to each other in a very firm manner; and the outer surface of the mass was so covered over by a new membrane, as to make its external appearance smooth and uniform. When this membrane was removed, and the adhesions were separated, which was done with difficulty, the coats of the intestine appeared to be quite healthy. The disease was evidently of very old standing, but the patient had never been known to complain of any uneasiness in the bowels till the fatal attack." 128.

From the number and variety of the cases now extracted, the reader will perceive how diversified are the pathological phenomena of ileus, and in what a variety of stages of action fatal cases may occur! Thus, death may happen during simple distention of the affected gut; or life may continue until the disease have advanced another stage, and exhibit the incipient fruits of inflammation; or inflammation may have been permitted to proceed to effusion of coagulable lymph; or the symptoms may continue till gangrene came on, and only terminate with the life of the intestine. In the first instance the disease seems to the Dr. to be little more than *paralysis* of the muscular coat; in the second, paralysis is complicated with inflammation: and to the third and fourth are superadded inflammation and its effects. But all are merely grades of one and the same disease, and, however their remote causes may differ, the effects produced by these are very much the same. In reviewing the cases above detailed, it will be seen that distention is the only uniform appearance which they present; but, whether this distention is to be considered as an effect or a cause, is, perhaps, a difficult but important question. The author's reasoning upon it is ingenious, and the evidence which he brings in favour of his views must be admitted to be strong where it fails to produce conviction. According to him the distended part is the seat of the disease, and the part which appears contracted is collapsed because empty; spasm is, therefore, wholly excluded, and his theory resolves itself into a mere loss of muscular action, or *paralysis*. But, lest we should misinterpret, or be misunderstood, we shall bring forward the language of the original on this point.

"It is probable, therefore, that there occurs in the state of ileus a certain loss of the muscular power in a portion of the canal, in consequence of which it does not act in concert with the other parts, but becomes distended by the impulse from the parts above, which in the healthy state would have excited it to contraction.

"In a fatal case of ileus, however, we generally find one part of the intestine in the state of distention here referred to, and another part empty and collapsed, presenting nearly the form of a cord; and there has been supposed to be a difficulty in determining which of these is the primary seat of disease,—some having contended that the collapsed part is contracted by spasm, and thus proves a source of obstruction, which leads to the distention of the parts above. The doctrine of spasm, as applied to this subject, must be admitted to be entirely gratuitous; and we must proceed upon facts, not upon hypothesis, if we would endeavour to throw any light upon this important pathological question. The following considerations seem to bear upon the inquiry:—

"1. The collapsed state of intestine, in which it assumes the form of a cord, appears to be the natural state of healthy intestine when it is empty. We often see nearly the whole tract of the canal in this state in the bodies of infants, who have died of diseases not connected with the abdomen, but in whom the bowels have been kept in a very open state up to the period of death. We cannot doubt that a similar state of uniform contraction is the healthy condition of other muscular organs when they are empty, such as the bladder. We have then no sufficient ground for assuming that the state of uniform contraction of intestine is a state of disease; on the contrary, the facts favour the supposition of this being its healthy condition when it is entirely empty.

"2. On the other hand, we learn from various cases, particularly from the remarkable case, (Case L.) that a state of uniform distention, with lividity, may occur as a primary disease of the intestinal canal, without any appearance of obstruction, and without any part of it being in a contracted state.

"3. In a case of ileus, the collapsed parts are almost invariably found in a healthy condition at all periods of the disease; the morbid appearances, whether inflammation, lividity, exudation, or gangrene, being almost entirely confined to the distended parts.

"4. In Case XXXV. every obstruction below was entirely removed, while the parts above were, to external appearance, in a healthy state,—and yet the action was entirely suspended.

"5. In Case LI, the cause, which uniformly acted in so singular a manner, must be supposed to have acted upon a part only whose action was impaired, not upon one which was spasmodically contracted.

"6. In Cases XXIX, XXX, and XXXI, we see the state of distention arising from causes entirely of a different nature, without the peculiar contraction here referred to; and on the other hand, in Cases XXXII, XXXIII, and XLIV, in which the disease was distinctly traced to a mechanical cause, this peculiar contraction existed below the seat of the obstruction, but could not be considered as having had any influence in producing the disease. In Case XXXIII, also it is to be remarked, that the contracted part was repeatedly and freely dilated during the course of the disease, without any effect in relieving the parts above.

"On these grounds, I submit the probability of the opinion, that, in a case of ileus, the distended part is the real seat of the disease; and that the contracted part is not contracted by spasm, but is merely collapsed, because it is empty, its muscular action being unimpaired." 136.

Now, although not disposed to travel to the same extent with the Dr. in his theory of ileus, nor prepared to criticise, *seriatim*, the arguments by which it is supported, we are ready to contribute our meed of approbation to its ingenuity, and unwilling to let it pass without some further notice.

That the distended intestine is generally in a diseased state is probable, and that this disease arises from want, or deficiency of muscular power, we believe;—that the contracted intestine may present no appreciable derangement is admitted, and that its diminished area may result from its being empty may occasionally be true; and, finally, that a distended condition of the fæcal tube *may* exist as a primary affection we consider proved by several of the facts and cases above recorded. But it must be equally certain, that ileus does arise from obstruction, and that distention may be the first effect of this obstruction. The cases of the impacted gall-stone and strictured colon will be regarded, we presume, sufficient vouchers for this point. Now, if obstruction to the passage of the fæces can and does occasion ileus, we would ask our author what evidence exists against the doctrine, that irregular muscular action, or, in other words, spasm, is never the cause of this obstruction? We are not aware that any of his cases or facts furnish such evidence, and we are certain that many of the causes and symptoms, and much of the treatment of this disease, are in favour of this view.

In the first place, attend to the nature of the causes;—they are generally such as are calculated to excite spasm, as cold applied to the surface of the body, acrid and poisonous matter received into the stomach and applied to the surface of the intestines, disordered bile, and unhealthy fæces. Secondly, examine the character of the symptoms; they are generally such as betoken the presence of spasm, as excruciating pain relieved by pressure, violent vomiting, tenesmus, and retraction of the muscles of the abdomen. The last symptom we should wish the Dr. to explain upon any other principle than spasm, and the first can be regarded as attending any disease rather than palsy. Thirdly, observe the remedies most effectual in relieving or removing the disease;—carminatives, opiates, tobacco enemata, cold affusion, and depletion. These are not such as would be first selected or most depended on in the cure of palsy, yet they constitute the sum and substance of our hope in ileus. In the fourth place, if a piece of distended intestine be the seat of ileus, and loss of muscular power be the cause of this distention, on what physiological or pathological principle will the Dr. account for this partial paralysis, and are the causes, symptoms, and progress of the disease, in appropriate keeping with its supposed nature? Is a sudden exposure to cold, or an unexpected suppression of the menstrual discharge, or the presence of acrid matter in the alimentary canal, among the most active causes of paralysis? Or is violent pain, or incessant vomiting, or tenesmus, or spasm of the abdominal muscles, or relief from pressure over the abdomen, among the most pathognomonic signs of palsy? Or is death in the course of a few hours or days, while nothing can be seen upon dissection, but a few alternate patches of contracted and distended gut, without vascularity, inflammation, effusion, or gangrene, the ordinary result after such a period in palsy? In the fifth place, what symptomatic or pathological difference appears between those cases which did arise, according to the Dr.'s own showing, from mechanical obstruction, and those which are supposed by him to have originated in partial palsy? Let cases 36 and 37, which were occasioned by strangulation of a part of the fæcal tube, be compared with cases 27 and 28, which are considered by the author as pure specimens of ileus from loss of muscular power; let their symptoms during life, and their appearances after death, be viewed in connexion, and then let the reader draw his own conclusion as to the *modus formandi* of the last two. During life we find violent pain of abdomen, urgent vomiting, and obstinate costiveness, the first, the last, and the leading symptoms of each; the treatment in all is alike, their duration very much the same, and the appearances of all upon inspection are inflammation and gangrene of the distended parts. And, lastly, we are not disposed to admit that a healthy intestine in an adult, when empty, and simply because it is empty, will always contract itself into an apparently solid cord; nor that a distended portion of intestine, and simply because it is distended, is always in a state of disease. Mere distention of the coats of an organ is insufficient to establish the presence of disease in these coats, in the absence of other less equivocal symptoms. Distention under such circumstances may be no more than excessive relaxation, and excessive relaxation is frequently an effort of nature to relieve disease, and not disease itself. Thus, spasm, or stricture of the urethra, may prevent the bladder from discharging its contents, and great distention of the organ is the consequence; but who will maintain that the bladder thus distended is in a diseased state? 'Tis true that this distention may occasion gangrene; but then the gangrene is a mere accident, which depends upon the degree of this distention. So is it frequently, we imagine, in the case before us;—a portion of the intestinal tube becomes obstructed, an impediment is thus presented to the progress of the fæces beyond the point of obstruction; but the peristaltic action from behind must occasion one of two results, either relaxation of the spasm, when the fæces shall be allowed to pass on, or distention of the tube adjoining the spasm, to give room to the fæces urged forward by the *vis a tergo*. The first result, we consider produced in those transient cases of colic which arise from sudden exposure to cold, are attended with severe pain, and last but a short time; the second result we regard as a secondary remedial effort following failure of the first, and except, perhaps, in a very few cases, not entitled to be called disease until overdistention destroy the organization of the parts, when inflammation, or other palpable consequences, will soon appear. Why is it that the distended part is always contiguous to the contracted portion? Is it not a too steady combination of circumstances to be explained by coincidence, that the distended and contracted parts should, in every instance, be placed in juxta position ? Since the distention is a primary affection depending on paralysis, and liable to occur in any portion of the tube, and since the contraction is a natural condition, depending merely on the absence of feces, which may be present or absent in any portion of the tube at the period of attack, these two states are merely accidents, which bear to each other no etiological relation, and have for their origin no common cause; yet, wherever there is contraction there is dilatation, and the dilatation immediately adjoins the contraction !

Did space permit, this theory ought to be more extensively discussed, but the few strictures now made will, perhaps, be sufficient to encumber it with as much *dead weight*, as shall prevent it from floating free and unexamined along the current of public opinion ; and as we wish, in every point of novelty and importance, to interest our readers as much as we feel ourselves interested, we shall not pursue this question further, but request them to take it up for themselves, convinced that whether they support or oppose the preceding train of argument, facts will be the only authority admitted in the investigation.

Before entering on the treatment of ileus our author very properly advises an examination of the abdomen, lest hernia be the source of disturbance; and, since a very small portion of strangulated intestine may give rise to a fatal attack of ileus, and this hernial state may escape even the suspicions of the patient, too minute attention cannot be paid to this point.

" In the medical management of cases which are referable to the general head of ileus, there are important distinctions to be kept in mind as to the state of the symptoms, which seem to require important diversities in the treatment.—It is impossible to delineate minutely all these distinctions, but there are certain leading varieties, which, in a practical point of view, may be briefly referred to. These are chiefly the following.

" 1. Obstinate costiveness with distention of the abdomen, and considerable general uneasiness, but without tenderness, and without much acute suffering.

" 2. The same symptoms, combined with fixed pain and tenderness, referred to a defined space on some part of the abdomen, frequently about the head of the colon.

" 3. Violent attacks of tormina, occuring in paroxysms, like the strong impulse downwards from the action of a drastic purgative,—the action proceeding to a certain point,—there stopping and becoming inverted,—followed by vomiting,—the vomiting often feculent.

" These forms of disease will be recognised by the practical physician, as constituting affections distinct from each other. In a practical view, the importance of the distinction consists in pointing at two modifications of the disease which seem to lead to differences in treatment ; namely, a state in which there is a deficient action of the canal,—and one in which there is a violent action limited to a certain part of it, though ineffectual for overcoming a derangement which exists below. The practical application of the distinction refers chiefly to the use of purgatives in ileus; and to the question, whether, in every case of ileus, the action of the canal requires to be excited by purgatives—or whether there are not modifications of the disease in which its action rather requires to be moderated. The adaptation of the remedies to the individual cases in fact demands the utmost discretion ; and it is impossible to lay down any general rules for it. There are some cases which yield at first to a powerful purgative, and there are others in which any thing like an active purgative is highly and decidedly injurious. A large dose of calomel will frequently settle the stomach, and move the bowels ; but, upon the whole, I think the best practice in general is the repetition, at short intervals, of moderate doses of mild medicine, such as aloes combined with hyosciamus. The peculiar and intricate character of the disease appears very remarkably from the fact, familiar to every practical man, that there are cases which yield to a full dose of opium, after the most active purgatives have been tried in vain. In regard to the use of purgatives, indeed, it may perhaps be said, that they form but a part of the treatment of ileus, and a part, too, which, in some forms of the disease, requires to be used with the utmost discretion." 143.

The cautious phraseology of this extract encourages the hope, that the Dr's. paralytic theory of ileus has not yet been enrolled among the sealed and settled articles of his creed. If this disease be so often paralytic in its nature, why should not purgatives be administered in the first stage before inflammation have come on?—They are stimulating, the disease requires stimuli, their stimulus is applied to the very part requiring it, and, in place of suspecting their adaptation, or questioning their efficacy, they should be faithfully and freely given. The truth is, that spasm, or inordinate action is in many instances the morbid cause; to administer severe purgatives would increase this action, and to increase this action would aggravate the symptoms; therefore, as the Dr., not very consistently, yet judiciously observes, "the best practice in general is the repetition, at short intervals, of moderate doses of mild medicine, such as aloes combined with hyosciamus." This is the very language of Hoffman and Cullen, and when the advocates of schools, so differently organized, agree in their practical directions, the theories advanced by either are of less importance. Speranza, who thought ileus depended on inflammation, recommends the lancet as the leading remedy; and, although proselytes to another faith, we attach much importance to judicious depletion. Violent and neglected spasm will soon pass into inflammation, and it is a fact of great practical value, that when inflammation supervenes on spasm, it is more inveterate in its ravages and more rapid in its course than perhaps under any other circumstances, and that if its approach be not prevented, it will often, after it has commenced, outrun the most vigorous remedies in its progress to gangrene. The tobacco injection is considered by the author the remedy of most general utility in all forms and stages of ileus. It is, certainly, a powerful medicine, and, if cautiously employed, may do much good. The quantity at first used should be small, increasing it according to the nature and degree of its effects. The application of cold to the lower extremities has been an old and favourite treatment, and its efficacy is attested by many cases. The patient is raised into the erect posture and cold water is dashed forcibly about his legs; some are satisfied with applying to the abdomen cloths wet with vinegar and water, and cold injections have been preferred by others. Cases do occur in which all these remedies may prove ineffectual; and recourse may then be had to opium; should abdominal tenderness, however, or an excited pulse betray the presence of phlogistic action, the lancet may be an appropriate preliminary. Lastly, stimulants will sometimes be found useful. When the constitution has been worn down by excessive pain, or the strength exhausted by active treatment, mild stimuli may and must be given. Aloetic wine, and tincture of aloes are highly spoken of, and a case is given which seemed to have been cured by galvanism. As this is a case of very considerable interest, and is referred to in corroboration of the Dr's. views of ileus, it will be proper to detail it; and the history, which succeeds, we adduce as a specimen of the extent to which this disease may proceed, while still within the influence of art and reach of recovery.

A Gentleman, aged, 50, laboured under pain in the right side of the abdomen, which felt hard, tender and distended, obstinate costiveness, and vomiting. The briskest purgatives were ineffectual, unless when aided by strong injections, and the relief they gave was very trifling. The application of galvanism was at length determined on, and its employment was almost immediately followed by a discharge of fæces, which was always preceded by a rumbling noise and commotion of the bowels. Twenty minutes was the period occupied by each application, but an urgent call to the night-stool not unfrequently broke up the operation. The stools were at first dark and hard, afterwards they became more natural, and in the space of ten days, all the tension and tenderness of the right side had completely disappeared.

A woman, aged 20, was attacked with ileus in so violent a form, that for the first five days nothing was done which gave relief. On the 6th, she had urgent vomiting, pulse was 120, bowels bound, abdomen generally tender. The lancet and other means were again tried without effect, and in the afternoon the surface became cold, the features collapsed, the pain ceased, the pulse 140, vomiting severe, and in short, she appeared moribund. A small glassful of wine was now given every hour, and after some time she rallied, when the tobacco injection was tried in small quantities frequently repeated, and, what few would have anticipated, the sinking was not increased but diminished by it, and the vomiting was materially reduced: —On the 7th, her symptoms were all decidedly improved, and she was free from pain; from this time convalescence proceeded steadily.

"The remedies which I have now mentioned are those of which I have most experience; but various others are to be kept in mind, as being sometimes useful. The warm bath is often beneficial at an early period of the disease, before there are any inflammatory symptoms. Crude mercury, in doses of one or two pounds, I have tried repeatedly, and in some cases it certainly appeared to allay the vomiting; I have not observed any other effect from it. The forcible injection of a large quantity of fluid, to the amount of six or eight pounds, is said to have been successful in some cases. In the Memoirs of the Medical Society of London, vol. ii. some interesting cases are described in which it was used with advantage. Large blisters over the abdomen are likewise extremely beneficial; also the oil of turpentine applied externally or by injection. When the vomiting is very urgent, so as to prevent medicines from remaining on the stomach, large doses of calomel, of from fifteen to twenty grains, often remain better than any other medicine, and even seem to allay the vomiting. In such cases, also, I have sometimes found benefit from giving powdered aloes, repeated at short intervals in combination with the oxide of bismuth. Whatever practice is employed ought to be zealously persevered in, notwithstanding the most unfavourable appearances; for the disease has been known to resist the most active remedies, and yet terminate favourably, as late as the 17th day." 147.

*Inflammatory Affection of the more external parts of the Intestines.* When commenting at the beginning of this paper on the Dr's general division of his subject, it was observed that he arranged inflammatory affections of the alimentary canal into three classes;—inflammation of its peritoneal, muscular, and mucous coats. These several forms he believes to exist separately, and to be distinguishable by one symptom, arising out of the varieties presented in the functional state of this organ. The truth and importance of two of these divisions,—affections of the mucous and peritoneal coats,—will be universally admitted; but we have already questioned the expediency of the third division, founded, as it is, on a single symptom of very variable character, and productive, as must be evident, of little, if any practical good. Without again going into the question, or formally extracting passages from the best writers upon it, in proof of the variable condition of the bowels as to function in inflammation of their mucous and peritoneal coats, we shall think it sufficient to appeal to the individual experience of our readers, whether they have realized in their experience the distinction, that when the peritoneum alone is inflamed the peristaltic action of the intestines is unaffected, while it is torpid and inefficient when the disease is confined to their middle coat? Although in general the Dr's. test, diarrhœa, applies to affections of the mucous lining, even in it the criterion is fallible; for it will be shewn hereafter that not only inflammation, but extensive ulceration and other disease may occur within it, without increasing in the least the action of the intestinal canal. As a mere nosological point, we may sanction this arrangement, believing that cases *may* occur in which disease is exclusively confined to *one* of these tissues, and as such an arrangement has no influence on the treatment. Indeed, its practical tendency the author himself gives up, by considering peritonitis and enteritis together, as either one and the same disease, or diseases so prone to pass into each other, that they are at last very generally combined.

*Peritonitis* is treated of in the present volume under three forms, *simple, erysipelatous,* and *chronic;* the first is conjointly described with *enteritis,* and the last two in distinct sections.

*In simple peritonitis* the patient complains of abdominal pain, which may be as variously seated, as it is different in degree. In some cases it is equally diffused over the entire cavity, at others it is confined to a single region, and occasionally it appears in a still more limited form, being comprehended within the site of a single organ and the space of a single inch. When inflammation of this membrane occurs near the kidney, the Dr. thinks ischuria renalis may be the consequence, and there is no doubt but that it often deranges the functions of those viscera which it approximates or attacks. When in the lower part of the abdomen the bladder sympathizes, when in the upper, the stomach, diaphragm and liver are deranged. The pain is much increased by pressure, inspiration, and any exertion which will agitate the viscera of this cavity; and it is a fact of considerable value, that in some cases of the most inveterate character, there is either slight uneasiness only, or the pain moves irregularly about, remaining for a few hours in one spot, and suddenly removing to another. At first the pulse seldom rises above its natural standard, but as the disease advances it becomes accelerated, and while generally small it is sometimes round and full. Tympanitis is a frequent symptom of the last stage; it seems to arise from loss of tone in the texture of the gut, and is a very unfavorable sign. Simple peritonitis may be fatal in three days, or, by passing into a chronic or less active form, may occupy several weeks, or even months, and upon dissection exhibit either serum, lymph, pseudo-membranes, or pus. Gangrene is conceived to be a rare result, except when the muscular tissue is implicated, and when it is met with it is slight, partial, and accompanied with extensive deposition. —This last sentiment, for reasons already given, is, we believe, insufficiently supported.

"Enteritis differs from simple peritonitis chiefly in the presence of vomiting and obstinate obstruction of the bowels. The pulse also is in general more permanently frequent, and the pain more violent and constant, often resembling the tormina of ileus. This, however, is not invariably the case; enteritis, on the contrary, being sometimes characterized chiefly by fever, with urgent vomiting and obstruction of the bowels, with tenderness of the abdomen, but without much complaint of pain. This variety seems to occur chiefly in young persons, as is exemplified in Cases LXIII, and LXIV. The pulse in enteritis is generally small and rapid, but not uniformly so, for we may find the disease with a full pulse and little increased in frequency, as in Case LX.

"Enteritis is generally fatal with a tympanitic state of the abdomen and rapid sinking, and we commonly find on dissection extensive deposition of false membrane, with adhesion, often combined with deposition of flocculent or puriform fluid, and generally lividity, or some degree of gangrene. The disease, we have reason to believe, consists in inflammation affecting both the peritoneal and muscular coats at once; and it is probable that it may supervene either upon ileus or peritonitis, or may take place at first in its complete form. We shall afterwards see cause to conclude that it may likewise supervene upon inflammation beginning in the mucous membrane." 155.

Out of a rich abundance of very interesting examples we shall select four cases, as illustrative of certain phenomena in the character of peritonitis, which the writer has laid down in the form of conclusions from the facts adduced.

"1st. Extensive and highly dangerous inflammation may exist in the intestinal canal without obstruction of the bowels, and it may go on to a fatal termination while the bowels are in a natural state, or easily regulated by mild medicines, through the whole course of the disease." 171.

A young lady, aged 20, was seized, on the 9th of June, with symptoms of peritonitis, which were so relieved by ordinary treatment, that on the 12th she appeared convalescent. But on the evening of that day a brisk dose of aloes and colocynth was taken, which caused much irritation, and on the 13th her disease retured. On the 14th, severe pain, and tenderness of bowels, and frequent pulse, were the leading features. During the day she was bled twice, after which the pulse became small and the aspect depressed, but the abdominal tenderness continued, yet without vomiting. Weak tobacco injections, cold, and blisters to the abdomen, with small doses of aloes and hyosciamus, gave much relief; the pulse fell, the tenderness subsided, the bowels wrought freely and without pain, and in three or four days convalescence was restored. About the 25th, a parotid swelling appeared, which suppurated and discharged a little matter by the ear; but the inconvenience it occasioned was trifling. On the night of the 2d of August she retired to rest, without any complaint except the parotid swelling, and early in the morning of the 3d, she awoke with cough and great dyspnœa. About 11 o'clock she was visited by the Dr. who found her sinking. Her face was cadaverous, breathing frightfully oppressive, frequent pulse, and her apartment was marked by an intolerable fetor. Thinking that the swelling of the parotid had burst into the larynx, it was opened; but healthy pus was discharged, and she died about 12.

*Inspection.* A pound of thin puriform matter, of intolerable fetor, was contained within a cyst of coagulable lymph, formed betwixt the diaphragm and upper surface of the liver; the left (right?) lung adhered to the diaphragm, and the diaphragm was perforated by a small opening, through which the matter from this cyst had passed into the bronchial tubes, and was traced to the trunk of the trachea. Peritoneal coat of liver inflamed, but internal structure quite healthy. Intestines universally adherent to the omentum and abdominal parietes, and among those adhesions were several cavities containing pus, one of which appeared to communicate with the great abscess above the liver. Behind the uterus two large abscesses were found in the pelvis. P. 162-3-4.

"2d. No diagnosis can be founded in such cases on the appearance of the evacuations. These may be slimy and in small quantity; they may be copious, watery, and dark coloured; or they may be entirely natural."

A gentleman, aged 25, was affected with pain of bowels, dysuria, and frequent desire to go to stool, with scanty slimy discharges. Pulse natural, several thin feculent stools were produced by oil, but the pain continued in paroxysms, for which sixteen ounces of blood were abstracted, and an opiate given. 19th. Passed an easy night, but in the morning the pain returned with increased violence, accompanied by dysuria and vomiting; pulse between 90 and 100, several feculent and consistent stools after a mild enema; 16 ounces of blood were again taken, aloes administered during the day, and an opiate at night. 20th. Much relief was again for a time obtained by the lancet, but sickness, depression, and faintness soon succeeded, his respiration became short, countenance anxious, voice feeble, and abdomen so tender that the pressure of the bed-clothes was intolerable; pulse 100. Was again bled from the arm and again relieved, took some aloes and had his bowels moved, breathed more freely, bore pressure over abdomen better, and, although on the night of the 21st he was delirious and vomited occasionally, from that time he improved, and ultimately got well. P. 160.

"3d. Extensive and fatal inflammation may be going on with every variety in the pulse. It may be frequent and small; it may be frequent and full; or it may be little above the natural standard, through the whole course of the disease."

A gentleman, aged 20, had pain in lower part of abdomen. Pulse 84, full, bowels natural. Was twice bled, and took opening medicine, which so improved his symptoms, that on the morning of the 7th he appeared free from complaint. In the afternoon some laxative, which he had taken, produced unusual disturbance, and at night he complained of a fixed pain in the upper part of the abdomen, shivering and heat, pulse 84. 8th. Pain of abdomen continues severe and fixed, repeated vomiting, and bowels obstructed, for which repeated bleeding and other remedies were ineffectually employed; pulse 96. 9th. Pain unabated, belly tympanitic but less tender, vomiting less severe, pulse 126; on the morning of the 10th he died.

*Inspection.* All the intestines distended and glued together by very extensive adhesions; omentum highly inflamed and adhering to intestines; lower extremity of small intestines and part of colon gangrenous, and an opening had formed in the appendix vermiformis, through which fæces had escaped into the abdominal cavity. P. 166-7.

"4th. Extensive inflammation may go on without vomiting and without constant pain; the pain often occurring in paroxysms, and leaving long intervals of comparative ease."

A woman, aged 40, from exposure to cold in November, became affected with pain in left side of abdomen, at first remitting but afterwards constant, which was considerably diminished by repeated bleeding and other ordinary remedies. She continued liable, however, to transient attacks, for which purgatives and opiates were successfully employed. After one of these attacks a hard swelling appeared in left side of the abdomen, the original seat of pain, which gradually increased, suppurated, and discharged a large quantity of pus. This abscess continued open by several orifices for six weeks, when the tumour subsided, and the openings healed. During all this time her bowels were unaffected and her stools natural, but she had emaciated much. As soon, however, as the discharge ceased, she began to improve, complaining only of occasional pains, which were relieved, as formerly, by purgatives and opiates. But, on the 5th of May, a violent paroxysm occurred that resisted every treatment, and she died on the morning of the 6th.

*Inspection.* The intestines almost universally adherent to each other by recent and old connexions; about the middle of the small intestines the gut was bound down to the spine, and its calibre was much contracted. Above this contraction the canal was dilated into a large sac, but there was no vestige of the abscess beyond its cicatrix, which nearly corresponded with the situation of this stricture.

The practical truth of the following advice we would strongly urge, as, were we unable to vouch for its accuracy from our own experience, the cases above given would be more than sufficient.

"Keeping in view these sources of uncertainty, our chief reliance, for the diagnosis of this important class of diseases, must be on the tenderness of the abdomen. This symptom should always be watched with the most anxious care, whatever may be the state of the bowels, or of the pulse, or the actual complaint of pain,—and though the tenderness itself should be limited to a defined space of no great extent; for, we have seen, that with every variety in these respects, a disease may exist of a very formidable character, and be advancing to a fatal termination. A certain degree of pain upon pressure we have found attending a merely distended state of the intestine; but this differs from the acute sensibility of peritonitis in such a degree, that an attentive practitioner can in general have no difficulty in making the distinction. When the tenderness exists without distention, as is frequently the case in the early stages of peritonitis, there can be no difficulty in the diagnosis." 172.

As simple peritonitis and enteritis are purely inflammatory diseases, the curative indications are plain, and the measures for accomplishing them few and obvious. If called in at the commencement the lancet is our first resource, and, unless the constitution or other circumstances be unfavourable, it should not be laid down until every dangerous symptom have been removed. As we have just seen, neither the pulse nor the pain, which are our ordinary guides in treating the phlegmasiæ, can be depended on. These facts should be ever present to the practitioner, and should induce him to look out for surer land-marks. Abdominal tenderness is a safer guide in the first instance, and, so long as pressure can produce any considerable uneasiness, it may be assumed as certain, that the disease as yet has not been fully overcome. We observe, in the *first instance*, with the view of supplying what seems to us an important omission of the author;—that when the disease has advanced far, without receiving or admitting of active measures, the nervous system becomes frequently more or less implicated, and the natural sensibility of the affected parts is more or less impaired. Hence does it happen that, in the advanced stages of enteritis, the hand may be applied with firmness without eliciting much if any complaint, giving rise to the deception that the strength of the inflammation has been broken, and that the symptoms neither sanction nor require much therapeutic energy. Liberal depletion should be at first resorted to, and persevered in until a decided impression mark the treatment; and a very valuable recommendation of the author, if observed,—to follow up a large detraction with small ones frequently repeated,—may save the patient much blood, and the practitioner much trouble. This plan has the advantage of preserving the effect at first made at the very least expence to the system, of never permitting the disease to rally, but when once *floored*, of keeping it ever under; whereas, according to the ordinary custom, the advantage gained by the first bleeding is lost or much diminished before a second is resorted to, and the foe, which had been nearly overpowered during the first onset, is foolishly vouchsafed a breathing time, during which his shattered forces are reunited, and return invigorated to renew the contest. "The inflammation of a vital organ should not be lost sight of above an hour or two at a time, until the force of it be decidedly broken; and, unless this take place within 24 hours, the termination must be considered as doubtful." There are several circumstances to be collected from the preceding cases which shew that purgatives, at least of a drastic character, are injurious, while the inflammatory action is unsubdued. In two instances a relapse was the consequence of their employment; and, although the medical world feel very differently upon this point, we cannot help agreeing with the author, that purgatives make no part of the treatment of the early stages of enteritis. If the bowels be much constipated, either a mild aperient, or an injection, or both, will certainly be necessary in any stage to prevent distention, and to remove what would increase the existing irritability; and, towards the close of the attack, this class of medicines may be moderately exhibited, to prevent or remove that tympanitic state of the intestines which so frequently follows an attack of enteritis. With this view, aloetic wine, or aloes and hyosciamus, aided by friction externally and injections of beef-tea with turpentine, or tincture of assafœtida, may be used with caution; and, if the strength be low, some wine or brandy, sulphate of quinine, and laudanum, will add to their effects. In recommending this course we must be understood to speak only of that form of tympanitis which depends upon relaxation of the alimentary canal, and *follows* inflammatory action. There is another variety which *accompanies* inflammation, and there is a third form which occurs in the *first* periods of the disease, subsiding as the inflammation is subdued.

The tobacco injection is as strongly recommended in enteritis as in ileus, as tending to move the bowels and allay vascular action; it possesses one property which entitles it to much notice, and is, perhaps, peculiar to itself;—it is equally adapted to almost every stage and state of the disease. Leeches are entitled to no confidence except when the affection is limited, or the strength unable to support constitutional measures, and blisters are only to be applied on the wane of the symptoms. In a considerable number of cases evident advantage has accrued from the application of cold, by covering the abdomen with cloths wet with vinegar and water, and injections of iced water have been plausibly recommended. After the inflammation has been subdued, the pulse not unfrequently retains an irritable character, is quick and agitated; in such instances digitalis may be given freely and with much advantage; and occasionally great weakness of the pulse and general powers, cessation of pain and coldness of the body unexpectedly appear, indicating the arrival of gangrene, while stimuli judiciously given often remove every unfavourable symptom. This fact is strikingly embodied in the succeeding case.

A man, aged 40, was affected with ordinary enteritis, for which he was very judiciously treated; but, on the fifth day of the disease, every symptom unexpectedly assumed a very unfavourable aspect. The pulse was 140, feeble and irregular, face pale, features collapsed, and entire body covered with cold perspiration. Wine was given in large quantities, to the extent of three or four bottles during the ensuing 24 hours. On the next day his pulse was 120, regular, and every other symptom ameliorated; on the third, his pulse fell to 112 and was strong; and in a few days more he was quite well. P. 176.

*Erysipelatous Peritonitis.* It has been long known that the mucous membrane of the nose and fauces may, as well as the external skin, be the seat of erysipelas, and that this disease may be indiscriminately propagated from either of these textures to the other; and it has been suspected by a few, that internal organs were not entirely exempt from it. Drs. Stevenson and Gibson have recorded cases confirmatory of this suspicion, and Dr. A.'s experience goes still farther in support of it. In the Spring of 1824, an epidemic of a peculiar character appeared in the Merchant's Hospital at Edinburgh. Its leading features were a slight erysipelatous affection of the throat, in some the internal fauces were studded with aphthous crusts, in others they were swollen, and in not a few the gums were spongy, and the lips encompassed with irritable ulcers. The larynx was unaffected in every case, by which peculiarity it was distinguished from *diphtherite*, and, excepting in two instances which proved fatal from the supervention of abdominal disease, the symptoms were mild, and little treatment necessary. In these two cases the patients were attacked in the same manner as their companions, and were steadily convalescing when pain of stomach, tenderness of abdomen, and serious constitutional disturbance suddenly supervened, and cut them off in a few days. On inspection nothing of importance was found out of the abdomen, which exhibited deposition of lymph on various parts of the intestines, and a considerable quantity of milky puriform fluid in the peritoneal cavity. While this disease was raging in the hospital, many cases of a similar nature occurred to the Dr. in his private practice. In these the internal fauces were of a dark red but not swollen, and this redness was interrupted by aphthous crusts. The lining of the nose in general became tender, and discharged a copious morbid secretion, and the inflammation ultimately came out upon the face, assuming the character of common erysipelas. Thus, we have in these cases the same kind of disease and the same form of action successively attacking different textures, yet so modified in its results from this difference, that in the fauces it terminates in aphthæ, in the nose in depraved secretion, and on the face in ordinary erysipelas. The two fatal cases, we presume, will render it probable that this erysipelatous action may invade the lining membrane of the abdomen, as well as that of the mouth, and that its symptoms, history and post-mortem products, may sufficiently distinguish it from *simple* peritoneal inflammation. The histories which follow are adduced as illustrations of the same doctrine.

A lady, aged 50, was seized with erysipelas of left leg, attended with much pain and swelling of the foot. After seven days the erysipelas of the leg subsided, leaving the foot unrelieved. But, upon the 8th, the foot also became suddenly well, and, within a few hours after, severe pain was complained of, first in the epigastre, and then in the lower part of the abdomen. This pain was not much increased by pressure, but she moaned much, was anxious and restless, pulse 100, bowels open. Bleeding, and the other usual means were ineffectually employed, her strength sunk, and she died about 24 hours after the metastasis.

*Inspection.* The upper half of the small intestines was of a dull leaden colour, the lower half of a very dark red, the whole much distended, and a considerable quantity of sanious fluid in the sac of the peritoneum. Besides these there were no other vestiges of disease.

A woman, aged 30, while convalescing from an attack of erysipelatous inflammation of the throat, took some laxative medicine which unusually annoyed her, and, shortly after, pain of the abdomen came on, attended with vomiting. Pulse frequent and small, countenance exhausted and skin cold. It was attempted to bleed her, but little blood flowed, blisters, opiates, and various injections were employed, and she died on the evening of the following day,—48 hours from the attack.

*Inspection.* Omentum inflamed, and at the lower part adherent for a few inches to the sigmoid flexure of the colon; intestines distended and of a livid dark colour, without exudation; peritoneal sac contained a considerable quantity of puriform fluid.

In this form of peritonitis many particulars may be pointed out which are tolerably characteristic. The symptoms are exceedingly insidious when their degree is compared with that of the action which they indicate;—they are unusually rapid in their progress;—they are generally connected with some external form of erysipelas;—they are marked from their first appearance by an extraordinary depression of the vital powers:—the pathology is equally peculiar;—the ordinary result is effusion of fluid, frequently without any, and seldom with much adhesive exudation;—this fluid is sometimes bloody serum, sometimes serum mixed with pus, and sometimes it is milky, containing shreds of flaky matter; the intestines are sometimes of a uniformly dark red, occasionally slightly vascular, and frequently free from every mark of disease; in one of the above cases the omentum was inflamed. The Dr. believes that women in the puerperal state are obnoxious to both varieties of peritonitis as now described, and that much confusion has arisen from writers upon this subject having neglected to draw between them a proper line of distinction. In the purely *acute* form, the ordinary treatment, if early and promptly applied, is sufficient, and the fatal cases present extensive pseudomembranous depositions and adhesions; but in the erysipelatous variety the symptoms are more insidious, and are attended by fever of a typhoid character.

"This affection runs its course with great rapidity: it does not yield to, or does not bear, the usual treatment; and it shows on dissection, chiefly extensive effusion of a sanious, milky, or puriform fluid, with much less adhesion than in the other case,—often with none; and frequently without any sensible change in the appearance or structure of the parts. There is little doubt that it is a contagious disease, or that it is capable of being conveyed from one woman who is affected with it, to another who is in the puerperal state. It appears as an epidemic at particular times, being very frequent and very fatal while it prevails; and erysipelas, or other affections of an erysipelatous character, have often been observed to be prevalent at the same time. Some of the cases which I have described under this section bear an evident resemblance to this formidable disease.

"This modification of peritonitis we have seen may be fatal without any remarkable change in the organization of the parts; and there is ground to believe, that, in some cases, it admits of a cure at an advanced period by the evacuation of the matter. In such cases, we have reason to conclude, that the inflammation had been resolved by the effusion, without leaving any injury to the organization of the parts. Several cases of this kind have been reported to me, in which, after symptoms of peritonitis, chiefly in the puerperal state, purulent matter either found a vent for itself through the parietes of the abdomen, or was evacuated by tapping, and the patients recovered. I have even observed some facts which induce me to believe that, in some modifications of this affection, a certain degree of peritonitis is resolved by effusion; that the effusion is afterwards absorbed, and that recovery takes place by a process of nature alone. This, of course, cannot be ascertained with certainty; but I have seen cases, with slight and obscure peritonitic symptoms, leave a tumefaction of the abdomen with much suspicion of effusion, which after some time entirely disappeared." 191.

*Chronic Peritonitis.* The Dr. considers chronic peritonitis a more frequent disease than has been hitherto suspected, and, therefore, dwells at some length upon its symptoms and history; but, after the large space which has already been devoted to this part of the work, we shall content ourselves with a very condensed abstract of its leading peculiarities, and with a case or two descriptive of its most important points.

There is generally pain in some region of the abdomen, which pressure or motion aggravates, and this pain may be permanent or periodical, general or limited; but occasionally the uneasiness felt is rather tenderness than pain, and sometimes there is no complaint but of distention and emaciation. The abdomen is usually fuller than natural, and often decidedly tympanitic; deep-seated spots of induration, which are tender to the touch, may be sometimes felt, vomiting frequently attends the other symptoms, and the state of the bowels is as variable as the character of their dejections. This disease may come on without any obvious cause; or it may succeed to more acute symptoms of the same nature, to external injuries, or such febrile diseases as measles or scarlatina. Although not common in childhood it frequently occurs in youth; in persons farther advanced in life it is often complicated with pulmonary disease. Its progress is marked by hectic symptoms, frequent pulse, and great emaciation; and, on dissection, adhesions between the intestines, sacs of purulent matter amid these adhesions, thickening of the peritoneum and alimentary canal, ulceration of the mucous membrane and disease of the mesenteric glands, are the most frequent relics of diseased action. If no treatment have been pursued during its first stage, medicine can do little when it is far advanced; and, as its symptoms at any period indicate neither much activity nor strength, topical bleeding, blistering, rest, and mild diet, comprehend a majority of the remedies on which we can depend.

The succeeding case is a faithful specimen of the silent progress which this disease pursues in its course to dissolution, and should of itself be a sufficient warning against its insidious character.

A lady, aged 16, without complaining of any particular pain or even uneasiness, had been for several weeks losing flesh and strength. Abdomen somewhat tympanitic, tongue foul, appetite impaired, pulse 120. Gentle laxatives, tonics and warm-bath were employed, and, after the continuance of the above symptoms for some time, seemed to produce improvement. Her appetite was much better, her strength and spirits improved, and she slept well. The abdomen, nevertheless, remained somewhat tympanitic, and her pulse ranged between 100 and 120. This imperfect improvement continued but a short time, for during the next month every symptom lapsed into its former state; yet, except occasional gripings, not even uneasiness was felt in her abdomen. Towards the close of this month vomiting and diarrhœa supervened, and, after being confined for two or three days only throughout her entire indisposition, she died.

*Inspection.* The viscera of the abdomen were so agglutinated by disease that one intestine could not be distinguished from another; in this diseased mass there were several cavities filled with purulent matter, and presenting on their internal surface scrofulous ulcerations; much purulent fluid in the pelvis; much disease of the mesenteric glands, and considerable enlargement of the liver.

The following case supervened on rubeola:—

A boy, aged 5, a few days after a mild attack of measles, began to complain of pain of belly, loss of strength and emaciation. Pulse small and frequent, skin dry and wrinkled, appetite impaired, bowels irregular, being sometimes loose, at others slow. Various modes of treatment were ineffectually tried, and he died at the end of three months from the first appearance of his symptoms. *Inspection.* Intestines so extensively adherent to each other, to liver, stomach, urinary bladder, and abdominal parietes, that they formed one amorphous mass of disease; liver slightly, and mesenteric glands much enlarged; large quantity of coagulable lymph in peritoneal sac; lungs studded with solid tubercles.

### Diseases of the Mucous Lining of the Intestines.

The pathology of the mucous membranes is encompassed with very great difficulties, and is very imperfectly understood. In muscular and serous tissues the symptoms of disease are less equivocal, by being more perfectly pronounced, the constitutional sympathy which their derangements occasion is more acute and extensive, the sources of fallacy are less numerous, the danger to be feared better understood, and the difficulties to be contended with, perhaps, more completely within the influence of medicine. But in affections, and even serious and protracted affections of the mucous tissues, more especially that portion belonging to the alimentary canal, how uncertain is our every guide, how random our every idea! Neither the pulse nor the pain, the structure nor the function, can be here depended on; and how often do we see patients, whose brains alone betray symptoms of disorder, and exclusively absorb the attention of their attendants, while their intestines upon dissection shew the only disease, and this disease even ulceration of their mucous lining! These difficulties are fully and faithfully portrayed by the author, and, while in some parts of the preceding pages, we have been reluctantly driven to the opposition bench, upon the present subject we can sit and vote together.

As to the symptoms attending disease of the mucous texture in the alimentary canal, we may have purging or constipation, and not only so, but purging may arise from the mere presence of acrid matter in the intestines, while costiveness may be connected with ulceration; the stools may be thin and feculent, scybalous and mixed with morbid secretion, mucous and mixed with blood, or membranous and mixed with pus;—there is generally more or less fever, but the pulse and skin may be unaffected;—the pain may be acute or chronic, constant or remittent, and the most severe pressure may elicit none;—vomiting often occurs and is often absent, and, unless in the early grades of the disease, when tenderness on pressure and a tendency to purge will be generally present, there is no one symptom so uniform or faithful as to be implicitly accredited.

The pathological products vary as much as the symptomatology;—portions of the mucous membrane may be somewhat red, or highly inflamed; its internal surface may contain flakes of coagulable lymph, or an entire lining of false membrane;—patches may be elevated above the common surface, and of a bright red;—small round or oval portions of it may assume a dark grey colour and pultaceous consistence, which, when separated, leave so many ulcers of the same size;—or these dark spots may be surrounded by areolæ of inflammation;—or the mucous surface may be studded with vesicles, containing clear fluid;—or a large portion of it may become soft, dark, and gangrenous; or it may exhibit ulcers of every form and variety, from a solitary and insignificant abrasion of surface to a deep and extensive destruction of substance with perforation; or, finally, firm tubercles may studd its surface, and pustules, like those in small-pox, have been discovered on it.

With symptoms so conflicting for our guide, and pathology so various for their elucidation, it cannot be considered strange that our knowledge of this tissue is defective, that writers estimate its derangements at very different rates, and that in many particulars some may materially disagree, and not a few be wholly ignorant. The present author has done much in this department; he has endeavoured to separate every accidental appearance upon dissection from what is purely the production of disease, and to classify these results according to their stage of maturity, or degree of importance. But he has done more; he has attempted, amid so much confusion and variety, to link certain appearances after death to certain symptoms during life, and thus make out an etiologico-pathological chart of all the diseases in this department of the mucous system. Thus, when this membrane is inflamed, the bowels, he says, are irritable, and the stools feculent and thin; when inflamed portions of it are raised above the neighbouring surface, if the disease be in the small intestines, the stools are bloody mucus mixed with scybala, if in the large intestines, with thin fæces; if the mucous lining be gangrenous, the dejections are dark and offensive, like the washings of putrid flesh; if it contain dark patches, surrounded by inflamed areolæ, or if these patches be dark, softened, and easily separable, the bowels are irritable, but the stools are tolerably healthy; if vesicles were found upon it, the stools consisted of tenacious mucus, assuming sometimes the form of tubes, and sometimes that of solid cords; if the membrane were ulcerated, and the ulcers in their earliest form, there would be a tendency to purging; if these ulcers were large with elevated edges and sloughing floors, copious discharges of muco-purulent matter mixed with blood, or shreds of flaky matter, are produced; and if they be large and deep, with elevated fungous edges and a dark fungous bottom, the intestinal functions and secretions may either be very much deranged, or perfectly natural. Although we believe that many of these statements, perhaps a majority, are founded in fact, we fear that in some of them the Dr. is either too learned to be instructive, or too fanciful to be depended on. In a great majority of the valuable cases with which this part of the work is enriched, the character of the tongue has been comparatively neglected; and when we see the minuteness of Dr. A.'s inquiries on other points, and reflect upon the delicacy of the probe with which he must have examined the most of the cases laid before us, we feel somewhat astonished, but more disappointed, that he should have so overlooked a source of light, which, in our estimation, is as steady in its occurrence, and as faithful in its information as any other upon which he has depended.

*Active* inflammation of the mucous lining may terminate in several ways, the most important of which are the following:—

"I. It may be fatal in the inflammatory stage,—a greater or less extent of the membrane presenting numerous patches of redness, which are in general sensibly elevated above the level of the surrounding parts; and in some cases, these elevated portions present on their surface numerous minute vesicles. These are most commonly observed in the disease as it appears in infants; and at a certain period of their progress, the vesicles seem to pass into minute ulcers.

"II. By gangrene, a portion of the mucous membrane appearing of a uniform black colour, and of a very soft consistence, in which the muscular coat in some cases appears to participate. Vesicles full of a putrid fetid fluid have also been observed upon the membrane.

"III. By ulceration of various extent and appearance, generally mixed with fungous elevations.

"IV. By passing into peritonitis or enteritis. This takes place in two ways. In the one, the inflammation seems to extend uniformly through the coats, until they are all affected; in the other, one of the ulcers perforates the intestine, its contents escape into the peritoneal cavity, and very rapid peritonitis immediately follows." 226.

Dysentery frequently proves fatal in the *first* way, and the following case terminated in *gangrene*:—

A man, aged 50, was seized with general uneasiness of the abdomen; on the 8th and 9th of October he passed several stools, which consisted principally of blood, after a dose of oil; on the 10th there was much tenderness of the lower belly, with dysuria, the stools were not, however, bloody, but watery, dark and offensive, like the washings of putrid flesh, and he died on the 16th. *Inspection.* The great intestines much distended; that part of them between the anus and middle of the arch of the colon gangrenous and black; from this to the caput coli they were variegated by numerous patches of a livid colour. The mucous coat, corresponding with the gangrenous part of the colon, was of a uniform black colour, soft and easily separated, and that lining the more healthy half was elevated into irregular patches of a deep red, with intervening portions in a more healthy state. On the inner surface of the caput coli were several distinct ulcers, and a few inches of the ileum beyond this were vascular and distended.

The *third* way in which this class of affections terminates is illustrated by the following history:—A girl, aged 3, about three weeks before her death was attacked with vomiting, pain of abdomen, and frequent calls to stool; her tongue was white, and she had much thirst. These symptoms continued until a few days before death, when oppression, aversion to exercise, screaming, and occasional coma came on; pulse from 130 to 150, stools frequent, green and yellow, pupils unaffected. *Inspection.* Ileum highly vascular, its mucous coat covered with many inflamed patches of a fungous nature, which were elevated and contained small ulcers; the portions of membrane intervening between these patches were healthy; mesenteric glands enlarged and vascular.

The *fourth* and last result of inflammation in this texture is secondary peritonitis. A woman, aged 38, who had been for more than a week labouring under fever, became suddenly seized, two days before dissolution, with most violent pains in abdomen and urgent vomiting, pulse 130, some hiccup. On *inspection*, the mucous coat of small intestines covered with small ulcers, one of which, seated in the lower part of ileum and surrounded by a ring of inflammation which was studded with small ulcers, had destroyed all the coats of the canal, and opened into the general cavity; peritoneal surface of small intestines much inflamed.

The following remarkable case we give, as containing within itself a specimen of nearly all the modifications of disease in the mucous membrane, and it is an important circumstance, that all this disorganization was the result of only nine days action. The reader will perceive how the character of the stools varied with the stage of the disease.

A lady, aged 35, on the 7th of July, was seized with vomiting, purging, and abdominal uneasiness, to remove which various means were tried in vain. On the 8th bleeding was attempted, but a very small quantity of blood was obtained; on the 9th, pulse 120, much vomiting, purging, and pain, tongue loaded, countenance anxious; bleeding, blistering, calomel and opium, and opiate injections, were used with very trifling relief, and she died without any remarkable change on the 15th. The stools during her illness were very various; at first they presented nothing different from those passed in diarrhœa; on the fourth day of the disease they were thin and mixed with bloody mucus; on the sixth they were fetid, and let fall, after standing for some time, some puriform fluid, very fetid and variegated with round spots of blood; and on the 8th and 9th they became dark, watery, and less feculent. On *inspection* the small intestines appeared externally healthy;—the colon was rather thickened and red, and at its sigmoid flexure there was some false membrane deposited;—mucous membrane of stomach and upper part of small intestines healthy;—that of ileum inflamed, the inflammation gradually increasing as it approached the cæcum; lining of caput coli very dark, and covered by well defined ulcers; farther along the colon these ulcers became scattered, and interspersed with dark fungoid elevations of the mucous membrane. In the arch of this gut the ulcers became small like split peas, isolated and surrounded by pale membrane;—in its descending side its entire surface was brown, spongy, and fungous, without ulceration, and its coats thickened, even in some parts cartilaginous; this state of disease continued into the rectum, and only ceased about two inches from the anus, which two inches even had not escaped, as they were considerably vascular.

Dysentery, tenesmus, scybala, and bloody mucus have been associated in the reasonings of medical men for centuries, as nearly inseparable, if not etiologically allied; but many facts and testimonies tend to shew that dysentery may appear with every form of stool, and without either tenesmus or blood. Sir J. M'Grigor observes that tropical dysentery is often much more like the diarrhœa than dysentery of Cullen, and it is certain that the parts concerned in the dysentery of Cullen and that of warm climates are as different as their symptoms. The rectum generally and sometimes the lower part of the colon are the seat of the nosologist's disease; while the entire colon and often a large share of the small intestines were concerned in the colonitis of Dr. Ballingall, and the tropical dysentery of Dewar, Annesley, and other writers.

Before leaving this part of our subject we may observe that infants, of five or six months old, are frequently attacked by acute inflammation of this membrane, and, although the disease in its general principles is the same with that occurring in adults, it merits special notice, from the difficulty of distinguishing it from the bowel complaints of children at the period of dentition.

*Chronic Diseases of the Intestines.* Acute inflammation of the lining membrane of the alimentary canal, we have already seen, may destroy life during the acute stage; or may end in gangrene, in simple ulceration, and ulceration combined with peritonitis. We now add that it may also end in chronic disease, which, by producing only trifling and transient disorder, may continue for a long time without destroying life. Occasionally, however, emaciation and weakness are soon induced, the mouth and fauces become raw and aphthous, a burning sensation is felt in the stomach, and severe hectic symptoms appear. The character of this second class of mucous diseases is as variable as the first; there is usually severe diarrhœa, continuing for a few days and alternating with costiveness, sometimes with vomiting; in some cases the appetite is good, but more commonly it is capricious; abdominal uneasiness, of some kind and to some extent, is a usual concomitant, which may be only elicited by pressure, or may amount to permanent pain; the stools may be bloody, purulent and feculent, or entirely made up of indigested food. The post-mortem results are equally diversified; we may find red patches of the membrane fungous, and raised above the common surface, or small defined and isolated ulcers, interposed by healthy structure; when the disease assumes either of these forms the bowels are purged and the stools fluid and offensive. Or we may have extensive tracts of ragged ulceration alternating with fungous elevations; or, finally, the outer coats may become thickened and indurated, contracting the area of the tube, or forming adhesions to neighbouring organs. When this form of disease occurs—

"There are copious evacuations of morbid matter from the diseased surface, which are sometimes puriform, and sometimes consist of a mixture of a tenacious puriform fluid, with mucous, or semi-gelatinous matter; and the whole is often deeply tinged with blood. According to the extent of the disease, this discharge may come off uncombined and in considerable quantities; or it may be mixed with healthy feces. When the disease is confined to the lower part of the colon or rectum, we may have the feces coming off in a solid, or even a hardened state, but generally mixed with more or less of the morbid discharge; and, at other times, we may have the discharge coming off in considerable quantities without any appearance of feculent matter. When the disease extends along the whole course of the colon, the feces generally come off in a liquid state, and, in this case, we may have the evacuations consisting sometimes of thin healthy feces, more or less combined with the morbid discharge; and, at other times, we may find the morbid discharge coming off without any appearance of feculent matter. When the disease is in the small intestine, we seldom see the peculiar discharge uncombined; it seems either to be in smaller quantity, or to come off so mixed with fluid feces as not to be easily distinguished. This form of the disease, in which the ulceration is confined to the small intestines, seems to be of frequent occurrence in phthisical cases; and indeed it appears probable, that, in some of these, it is the primary disease, and that the affection of the lungs takes place at a subsequent period." 252.

The phenomena attached to all these varieties of chronic disease the reader will admit to have been abundantly diversified and sufficiently uncertain, yet there is still another form, which the Dr. has considered important enough to receive distinct consideration in a separate section, in which the symptoms are much more equivocal, in which, in truth, there are no symptoms at all.

The two following cases will serve as specimens:—

A gentleman, aged 60, who had enjoyed excellent health, if we except habitual costiveness, was reading to some friends after dinner, when he suddenly complained of a violent pain in the lower part of the abdomen, and vomited repeatedly. His countenance became pale, hands cold, and pulse feeble; the most judicious treatment was pursued without benefit, and he died in about six hours.

*Inspection.* Much feculent matter in the cavity of the abdomen, which had escaped through a hole in the colon, a little above its junction with the rectum; this hole was larger than a shilling, and was encompassed by a mass of indurated substance. Below this opening the intestine was very much contracted, and above it its internal surface was extensively ulcerated.

A gentleman, aged 35, became affected with symptoms of continued fever, which for the first two days were mild and excited no apprehension, but on the third, while at stool, he voided a very large quantity of fluid blood, fainted, and, upon recovery, again discharged so much as to saturate the mattress and carpet on which he lay. A variety of means were used to arrest it, but he died in a few hours.

*Inspection.* Intestines externally healthy, except lower part of ileum, which contained a patch of a very dark colour, that was much thickened; on the inner surface of this patch was a deep ulcer, as large as a shilling, with elevated edges, which was partially filled with fungus and coagulated blood.

Arterial hæmorrhage sometimes takes place from the rectum, and produces very great exhaustion. The constitutional symptoms it occasions are sudden and severe, the pulse soon becomes weak and rapid, the face pale, and vertigo, head-ache, palpitation, and restlessness are felt on the least exertion. On examination in such cases a small fungous mass will be discovered within the verge of the anus, on the apex of which a minute artery bleeds *per saltum.* The treatment is simple;—the vessel has only to be tied, and, should a ligature not effectually arrest the hæmorrhage, a free application of lunar caustic will add to its effect.

Before going into the treatment of these various affections, it may be well just to allude to one point, which has been much agitated, and about which much unsettled opinion yet exists;—whether ulcers of the intestinal canal heal, and, if they do, whether the mucous membrane be re-produced? The first of these points, the cases which we shall adduce would answer in the affirmative; the second, we fear, as far as the volume before us is concerned, must remain undetermined. That ulcers of the fecal tube may heal, as well as discontinuations of surface in any other part of the body, we know of no reason for disputing; 'tis true, that their situation and other circumstances will generally retard and often prevent the healing process, and that these ulcers frequently occur in connexion with general diseases of a severe and inauspicious nature; but such facts will only prove the difficulty, not the impossibility of their cure. That the mucous texture may be re-produced, we, a priori, see no reason to deny; but we have never witnessed such an occurrence, and excepting the testimony of Bright, and, perhaps, two other pathologists, we know of no facts nor cases to be depended on in favour of it.

A lady, who was of a feeble and delicate habit from her youth, fell after puberty into a decided state of bad health. In Winter she was afflicted with cough, diarrhœa, and pain of bowels; in Summer these symptoms abated, but never wholly disappeared. Large doses of muriated tincture of iron and hyosciamus seemed for a time to afford some benefit, but, after being afflicted with more or less of these complaints for several years, she died of phthisis at the age of 24.

*Inspection.* Lungs extensively tubercular, with numerous vomicæ; lower half of stomach thickened and contracted; several portions of the inner surface of the intestines too vascular, and in many places there were "small circumscribed smooth spots, which had every appearance of the cicatrices of ulcers, which had healed;" other viscera healthy.

A lady, aged 18;—whose case is given at very great length, but whose prominent complaints for more than twelve months were, at first, impaired appetite, disordered bowels, feeling of heat in epigastre, weakness and occasional headache; afterwards were added aphthæ in throat, tender state of tongue, thirst and general fever; and, ultimately, a daily fit of febrile symptoms which continued for several hours, cough, tubercular expectoration, pain in left side of chest, imperfect respiration and other symptoms of confirmed consumption;—at length died, and upon *inspection* air and much sero-purulent fluid were found in the left pleural cavity; left lung partly hepatized, partly infiltrated with pus, and in many places filled with tubercles. By an aperture through the pleura covering its *middle* lobe (was it the *right* lung?) an irregular cavity in its substance communicated with the pleural cavity, and there were several smaller chambers communicating with it. Right lung healthy. The mucous membrane of the colon was found thickly covered "with small spots of a clear white colour, which were remarkably distinguished by their colour from the mucous membrane surrounding them. Few of them were larger than the diameter of large pin-heads, and on minute examination they were distinctly ascertained to be vesicles, very little elevated, but when punctured discharging a small quantity of clear fluid. The whole surface of the membrane presented a very peculiar appearance, from the immense number of these spots with which it was covered, but the other coats were entirely healthy. In the mucous membrane of the caput coli there were two distinct spots of ulceration." P. 296.

In active diseases of the alimentary canal much depends upon prompt and early measures, and, seeing that the symptoms require from their obscurity unremitting attention to render them available in practice, no source of information should be left unexamined, in order to discover the seat and nature of the affection to be removed. To subdue the inflammatory action, to quiet the irritation, and to correct the morbid secretion of the canal, will comprehend all the treatment necessary in any of the preceding numerous varieties.

"I. For answering the first of these indications, the remedies on which we rely, when the case is seen at a period adapted to the use of them, are general and topical blood-letting, blistering, diaphoretics, and antiphlogistic regimen. On this subject, on which my own experience has been limited, I may now refer to the best practical writers on dysentery, as it is seen in various parts of the world, particularly Dr. Ballingall, Dr. Bampfield, and many others; and the practice has also received the high sanction of Sir James M'Grigor, under whose instructions it has become the established treatment of dysentery by the medical department of the army. Dysentery, indeed, may exist in a degree in which it may get well without bleeding, but so also may peripneumonia or bronchitis; and it may occur in unhealthy debilitated subjects, or in combination with low malignant fever, and may thus not admit of active treatment; but these circumstances only introduce new difficulties in regard to individual cases, and do not affect the general principles which regulate the treatment of the disease.

"The use of general bleeding must of course be regulated by the activity of the symptoms, the constitution of the patient, and the period of the disease; for it is probable, that in general the period for active treatment is soon over. Much benefit is often derived from free local bleeding, which may be accomplished by leeches, applied either to the abdomen, or, when the disease is seated in the lower part of the bowels, to the verge of the anus. As diaphoretics, the best is perhaps Dover's powder; Ipecacuan in powder, in doses of gr. i. or ii. three or four times a day, has also been much recommended, and James' powder, given in the same manner; but in all inflammatory affections of the mucous membrane of the intestine, the effect of antimonial preparations would appear to be rather questionable.

"II. The second indication, which is to quiet the general irritation of the canal, will be chiefly answered by mucilaginous articles and opiates, particularly Dover's powder, perhaps combined with chalk, with the mildest kinds of farinaceous food in very small quantity; and I imagine that much will be gained in the early period of the disease, especially when the affection is extensive, by taking into the stomach as little as possible of either food or drink; as from the morbid irritability of the parts, the mildest articles often produce great irritation. Suet, dissolved in milk, has been much recommended; and a favourite remedy in the time of Sir John Pringle was a combination of yellow wax and Spanish soap, melted together over a gentle fire, and then rubbed up with water. The warm bath is often beneficial, and equal gentle pressure of the abdomen, by a roller of elastic flannel, is a remedy which has been strongly recommended as of much efficacy in all stages and forms of dysentery.*

"III. For correcting the morbid condition of the membrane, after the force of the inflammatory symptoms has been subdued by the necessary means, various remedies appear to be useful, in different states and different stages of the disease. In the earlier stages, benefit is frequently obtained from doses of Dover's powder, of from 5 to 10 grains, combined with 1 grain of calomel, repeated, at first, every four or five hours, and afterwards at longer intervals. This applies to the dysentery of this country; in the more severe cases, which occur in warmer climates, Dr. Ferguson has strongly recommended a grain and a half of calomel, with one grain of ipecacuan, to be repeated every hour until the mouth is affected, when, he says, the dysenteric symptoms always cease. In the dysentery of tropical climates, calomel is given in still larger doses, as from 10 to 15, or even 20 grains, repeated three or four times a-day, generally combined with opium. Of this mode of treatment, as applied to the dysentery of tropical climates, I would not presume to give an opinion, because I have had no experience; but when I have seen a similar practice attempted in the dysenteric affections of this country, it has appeared to be decidedly injurious; and when mercury is given, it appears that the small doses of calomel combined with Dover's powder, in the manner which I have mentioned, is the form best adapted to the earlier stages of the disease. In a more advanced stage, when the morbid secretion continues after the inflammatory symptoms have been subdued, various remedies of a tonic and astringent nature appear to be useful,—such as, cusparia, lime water, oxide of bismuth, nitric acid, sulphate of alum, logwood, balsam of copaiva, acetate of lead, and various combinations of these with each other, and with small opiates, especially a strong decoction of cusparia with nitric acid and laudanum; and oxide of bismuth with cusparia and Dover's powder. Charcoal has been strongly recommended, and, in one very severe case, in which it was given in combination with Dover's powder, it appeared to Mr. Gillispie and myself to be decidedly useful. Nitric acid, combined with opiates, I conceive to be a remedy deserving of much attention even in the earlier stages, after the necessary evacuations.

"When the disease is chiefly seated in the lower part of the colon and rectum, various substances may be given in the form of injections. Of these, the most useful seem to be, in the early stages, mucilaginous articles, or thin arrow-root, with an opiate, an infusion of tobacco, or an infusion of Ipecacuan. After the first urgency of the inflammatory state has been subdued, I have seen decided benefit in relieving the tenesmus by injections of lime water, at first diluted with equal parts of milk or thin arrow-root, and with the addition of an opiate." 280.

Purgatives are very doubtful medicines in this class of diseases; if there be much irritability they will do harm; and if the bowels are loose they cannot be required. Constipation occasionally occurs, and in such cases they may be necessary; but the mildest must be chosen, and even these must be given in the mildest doses.

"In the chronic form of the disease, the morbid conditions which we have chiefly to contend with are either the chronic fungoid inflammation, or ulceration. The treatment is extremely precarious, and but few of the cases comparatively do well. The remedies which appear to be most generally useful are the following: lime water; vegetable bitters and astringents, especially the cusparia and logwood; preparations of iron; small quantities of mercury with opium, especially calomel with Dover's powder, or small doses of calomel with

* "See Dewar on Dysentery."

opium and ipecacuan; the resins, as turpentine, balsam of copaiva or tolu, with small opiates; sulphur with opium; nitric acid; various combinations of these remedies with each other, as a strong decoction of cusparia with nitric acid and laudanum. Repeated blistering on the abdomen is often very beneficial, also bandaging with a broad flannel roller, and tepid salt water bath. Sulphate of copper has lately been recommended by Dr. Grenville in various protracted affections of the bowels; and in any trials of it which I have had an opportunity of making in this class of diseases, it appears to be a remedy deserving of much attention. It is given in doses, at first, of half a grain, combined with an equal quantity of opium, and is gradually increased, if necessary, sometimes to the extent of gr. iii. with half a grain or a grain of opium, three times a day.

"In the treatment of all the affections of this class, much depends upon the most rigid attention to diet. Animal food in every form seems in general to be hurtful; and the greatest benefit results from a diet strictly confined to farinaceous articles and milk." 286.

Disease of the *mesenteric glands* is a frequent accompaniment on affections of the intestines, whether chronic or acute. At the earliest period of their disease these glands, when cut into, appear of a pale flesh-colour, and are somewhat enlarged; their texture then becomes firmer, and, losing its fleshy aspect, assumes an opaque white structure like that of tubercle, which softens down first into a cheesy consistence, and afterwards into a mass like ill-conditioned pus. When one of these glands, in the first stage of alteration, is plunged into boiling water, its fleshy colour is exchanged for an opaque white, and, when boiled for some time, its weight diminishes, and a residuum is left, which is of an opaque white, very firm and has the general characters of concrete albumen. In their more advanced stages of disease the loss occasioned by boiling is diminished, and the opaque white tubercular matter, when obtained pure, scarcely loses any. The Dr. thinks that during these changes albumen is deposited, at first in a soft and afterwards in a concrete state; and that the peculiar character of glands, affected with tubercular disease, depends on the presence of unorganized albumen in a very concrete form.

We have now closed a very long review of a very valuable work, and, although we have endeavoured to condense into our pages a great mass of important matter, we feel that our author has not yet received justice, and that our readers are not even yet in complete possession of the facts contained in this performance. But he, who bears an honest attachment to his profession, will not rest satisfied with the review, no matter how laboured, of a work so distinguished as the present; and he, who merely wishes to know the character and rank of a new publication, would, probably, give us little credit for carrying our labours any farther. Had the Dr. paid somewhat more attention to his style, he might have been throughout more elegant, and sometimes more correct. *Lapsus calami* are not uncommon, clumsy phraseology occasionally occurs, and there is a general looseness, both in the selection of his language and in the texture of his composition, which, in the estimation of some, may detract from the general ability of the work, and which a writer of Abercrombie's calibre ought to have avoided. These, however, are such trifling mar-strokes in a painting of such general excellence, that, if they do not tend by their *shade* to give vigour to the *light* of his happier touches, they can scarcely be considered to diminish the accuracy of the piece; and, while we believe a hint will not be thrown away, we feel any thing but a disposition to embody our strictures with the formalities and size of direct criticism.

